# Immune Activation and Anemia Are Associated with Decreased Quality of Life in Patients with Solid Tumors

**DOI:** 10.3390/jcm9103248

**Published:** 2020-10-12

**Authors:** Patricia Kink, Eva Maria Egger, Lukas Lanser, Michaela Klaunzner, Bernhard Holzner, Wolfgang Willenbacher, Maria Theresia Kasseroler, Dietmar Fuchs, Günter Weiss, Katharina Kurz

**Affiliations:** 1 Department of Internal Medicine II, Medical University of Innsbruck, 6020 Innsbruck, Austria; patricia.kink@tirol-kliniken.at (P.K.); eva.egger@tirol-kliniken.at (E.M.E.); lukas.lanser@i-med.ac.at (L.L.); guenter.weiss@i-med.ac.at (G.W.); 2 Department of Psychiatry II, Medical University of Innsbruck, 6020 Innsbruck, Austria; michaela.klaunzner@student.i-med.ac.at (M.K.); bernhard.holzner@tirol-kliniken.at (B.H.); 3 Department of Internal Medicine V, Medical University of Innsbruck, 6020 Innsbruck, Austria; wolfgang.willenbacher@tirol-kliniken.at (W.W.); marie-therese.kasseroler@tirol-kliniken.at (M.T.K.); 4 Oncotyrol Center for Personalized Cancer Medicine, Medical University of Innsbruck, 6020 Innsbruck, Austria; 5 Institute of Biological Chemistry, Biocenter, Medical University of Innsbruck, 6020 Innsbruck, Austria; dietmar.fuchs@i-med.ac.at

**Keywords:** immune activation, anemia, tryptophan, quality of life, depression, cancer

## Abstract

Anemia often coincides with depression and impaired quality of life (QoL) in cancer patients. Sustained immune activation can lead to the development of anemia. Furthermore, it also may go along with changes in tryptophan and phenylalanine metabolism. The aim of our pilot study was to study the relationship between anemia, immune-mediated changes in amino acid metabolism, and the QoL and mood of cancer patients. Questionnaires to measure QoL and depression were completed by 152 patients with solid tumors. Hemoglobin, parameters of immune activation as well as tryptophan and phenylalanine metabolism were determined in the patients’ sera. Anemic patients (51.7%) presented with higher inflammatory markers, and a higher tryptophan breakdown with lower tryptophan concentrations. They reported an impaired QoL and had higher depression scores. Patients with an impaired QoL (65.8%) also suffered from more fatigue and impaired physical, emotional, and social functioning. They, furthermore, presented with higher concentrations of inflammatory markers (C-reactive protein (CRP) and neopterin) as well as higher tryptophan degradation (in men) and higher phenylalanine concentrations (in women). Sixty-one patients (40.1%) had (mostly mild) depression. In these patients, a higher degree of Th1 immune activation was found. The results of our study suggest that cancer-related anemia goes along with an impaired QoL, which is also associated with immune-mediated disturbances of tryptophan and phenylalanine metabolism.

## 1. Introduction

Anemia is a comorbidity frequently encountered in patients suffering from malignant disease [1]. It is a condition characterized by fatigue, weakness, shortness of breath, or dizziness. All these symptoms impair the quality of life (QoL) of cancer patients significantly. Additionally, a high percentage of patients also have depressive symptoms [2,3,4]. In general, about 10%–25% of cancer patients suffer from depression [4]. However, depending on the type of cancer, the incidence of depression varies greatly [5]. Comorbid depression also goes along with increased functional and emotional impairment and reduced optimism regarding the effectiveness of the medical treatment [6,7]. It is often not recognized and, thus, not treated [8,9]. Furthermore, studies suggest a correlation between more severe depression and more rapid tumor progression as well as increased symptoms of cancer [10,11]. Depression and a decreased QoL have earlier been associated with decreased hemoglobin concentrations and increased levels of inflammatory markers [2].

In fact, in patients with malignant disease, anemia is mostly due to anemia of chronic disease [12]. Chronic inflammation with an enhanced release of pro-inflammatory cytokines induces disturbances of iron metabolism and, subsequently, a decrease of hemoglobin. However, the development of anemia might not only be due to a decreased supply of iron, but also diminished concentrations of the essential amino acid tryptophan might impair erythropoiesis [13].

Tryptophan (Trp) is important for protein biosynthesis (including hemoglobin synthesis) and several physiological processes including erythropoiesis. Furthermore, it is the precursor of the neurotransmitter serotonin (5-hydroxy-tryptamine, 5-HT) [14,15,16]. Within T-helper cell type 1 (Th1), immune response to the pro-inflammatory cytokine interferon-γ (IFN-γ) induces the enzyme indoleamine 2,3-dioxygenase-1 (IDO) to degrade tryptophan to kynurenine (Kyn) [17,18]. The extent of tryptophan breakdown can be assessed by the kynurenine to tryptophan (Kyn/Trp) ratio, which is a good marker for IDO activity [19]. In patients with malignant-disease-enhanced immune-mediated tryptophan, breakdown leads to decreased tryptophan levels [20]. Low tryptophan availability inhibits the proliferation of T-lymphocytes (and, thus, cellular immune response), but also impairs erythropoiesis [21] and influences patients’ mood [22,23]. 

Earlier studies proposed that inflammation and immune-mediated degradation of tryptophan are involved in the development of neuro-psychiatric symptoms and depression [24,25,26,27]. Elevated concentrations of the inflammatory marker neopterin, which reflects the extent of Th1 immune response [28], and a high Kyn/Trp ratio have been demonstrated to correlate with fatigue and depression in patients with malignant disease [29]. The accumulation of neurotoxic metabolites of kynurenine like 3-hydroxy-kynurenine (3-HK) and quinolinic acid (QUIN) might be involved in the development of neuro-psychiatric symptoms, but also a decreased formation of serotonin could play a role [30,31,32,33]. 

In addition, IFN-γ enhances the oxidation of tetrahydrobiopterin, which is an essential cofactor for the formation of the catecholamine precursor tyrosine (Tyr) from phenylalanine (Phe) via the phenylalanine hydroxylase (PAH), thus, reducing the concentrations of tyrosine [34]. Actually, impaired phenylalanine conversion was shown to be associated with the development of mood disorders [35]. Thus, immune activation might affect a patient’s mood and QoL via different pathomechanisms: by the development of anemia and also by inducing changes of amino acid metabolism [13,36]. Additionally, anti-tumor therapy might impair QoL and contribute to the development of depressive symptoms. 

Considering the link between chronic immune activation and both anemia and alterations of amino acid metabolism, it was the aim of our pilot study to characterize these relationships in patients with solid tumors more exactly. Furthermore, we wanted to find out whether immune-mediated changes of tryptophan and phenylalanine metabolism and anemia were associated with the patients´ physical and emotional functionality and whether anti-tumor therapy influences all these parameters. 

## 2. Materials and Methods

### 2.1. Study Population

We recruited 152 patients (44 female, 108 male, mean age 61.2 ± 11.4 years) suffering from solid tumors from the Department of Hematology and Oncology at the University Hospital of Innsbruck, Austria. 

Patients with an acute infection, getting immunosuppressive therapy, with depression, under treatment with antidepressants, or participating in another clinical study were not included in the study. Blood was taken from patients at admission (i.e., before the administration of chemotherapy or any other treatment). Patients who did not meet exclusion criteria, gave informed consent, and were treated at the Department of Hematology and Oncology of the Innsbruck Medical University from January 2014 to June 2018 were enrolled (but only once—we did not include consecutive samples and questionnaires of patients who came for chemotherapy, operations, or radiotherapy before participating in our study). Exact data regarding the exact diagnoses and the pre-treatment of patients are shown in Table 1.

The study conformed to the ethical principles outlined in the Declaration of Helsinki and was approved by the ethical committee of Innsbruck Medical University (ID of the Ethical vote: UN5222; session number: 329/4.6). All patients signed a written informed consent after an in-depth explanatory interview before participating in the study.

### 2.2. Assessment of Quality of Life and Depression

To assess the patients’ QoL the European Organization for Research and Treatment of Cancer Quality of Life Questionnaire Core 30 (EORTC QLQ-C30), version 3.0 was administered [37]. This 30-item questionnaire measures the patients’ functional status (15 items) and the occurrence of cancer-related symptoms (13 items) on a scale from 1 (“not at all”) to 4 (“very much”). It also asks the patients to self-rate their global health status and quality of life on a scale from 1 (“very poor”) to 7 (“excellent”). The raw data are subsequently transformed into six single- and nine multi-item scales, each with a range from 0 to 100 points. As a result, high scores indicate high global quality of life, high functionality, and a high symptom burden in the symptom scale, respectively [38]. 

To evaluate the extent of depressive symptoms, patients also completed the Beck’s Depression Inventory-II (BDI-II). It is comprised of 21 items with response options from 0 (low symptom manifestation) to 3 (high symptom manifestation). Total BDI-II scores range from 0 to 63 points and depression is categorized into “no depression” (0–8 points), “minimal” (9–13 points), “mild” (14–19 points), “moderate” (20–28 points), and “severe depression” (29–63 points). 

### 2.3. Laboratory Examinations

Within the scope of routine blood tests at admission (and before administration of chemotherapy), venous blood samples were taken and stored at −20 °C until analysis. Serum concentrations of the inflammatory markers C-reactive protein (CRP), leukocytes (WBC), and neutrophil granulocytes, as well as hemoglobin concentrations, were determined in the scope of routine laboratory examination. Patients were classified as being anemic if their hemoglobin concentrations were lower than 120 g/L and 130 g/L for women and men, respectively. Neopterin concentrations were measured by ELISA (Brahms GmbH, Henningsdorf, Germany). Tryptophan, kynurenine, phenylalanine, and tyrosine were determined by high-pressure liquid chromatography in the serum as described elsewhere [39,40]. The kynurenine/tryptophan (Kyn/Trp) ratio and the phenylalanine/tyrosine (Phe/Tyr) ratio as indicators for the activity of IDO and PAH, respectively, were calculated. 

### 2.4. Tumor Classifications

Patients were staged according to the Union for International Cancer Control (UICC) classification. UICC stages I and II are comprised of tumors that are limited to the organ of origin with extension T1 or T2 respectively. Tumors with UICC stage III can be either limited to the organ of origin with extension T3 or locally spread to regional lymph nodes (N1) with extension T1, T2, or T3. Finally, UICC stage IV is comprised of tumors that are limited to the organ of origin with extension T4, locally spread to regional lymph nodes (N1) with extension T4 or spread to more distant lymph nodes (N2, N3) or other organs (M1) with any extension [41].

### 2.5. Statistical Analysis

Quantitative variables are expressed as medians (25th, 75th percentile) if they showed no normal distribution in the Kolmogorov–Smirnov test. Categorical variables are expressed as prevalence and percentage. To compare variables between groups, a Mann–Whitney U test or Kruskal–Wallis test was employed. Group differences in qualitative variables were assessed by Chi-square or Fisher’s exact test. Spearman rank correlation or partial correlation analysis was used to examine associations between variables. All tests used were two-tailed and *p*-values < 0.05 were considered as statistically significant. The statistical analysis was performed with IBM SPSS Statistics, Version 25 (IBM Corporation, Armonk, NY, USA).

## 3. Results

Information on the type, stage, and treatment strategies of cancer, general patients’ characteristics, and laboratory measurements for the whole patients’ cohort and separately for women and men are shown in Table 1.

### 3.1. Anemia Coincides with Immune-Mediated Tryptophan Degradation

**Figure 1:**
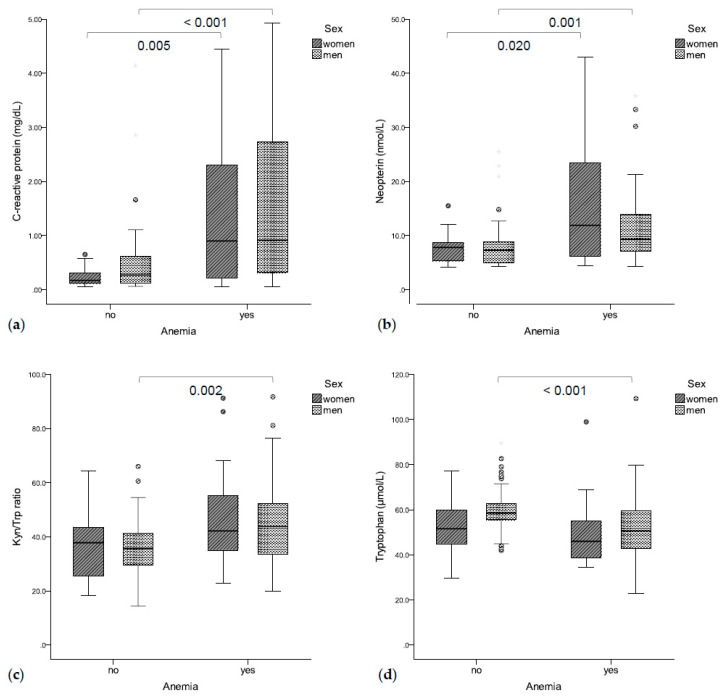
(**a**) Anemic patients had significantly higher C-reactive protein (**b**) and neopterin concentrations. (**c**) Anemic men also had a higher Kyn/Trp ratio (**d**) and lower tryptophan concentrations.

Seventy-eight patients (51.3%, 21 women, 57 men) suffered from anemia. Anemic patients presented with significantly higher CRP (0.90 mg/dL vs. 0.26 mg/dL, *p* < 0.001, Figure 1a) and neopterin concentrations (9.5 nmol/L vs. 7.4 nmol/L, *p* < 0.001, Figure 1b) as compared to non-anemic patients. Accordingly, hemoglobin concentrations were correlated with CRP (rs = −0.450, *p* < 0.001) and neopterin concentrations (rs =−0.404, *p* < 0.001). Additionally, higher Kyn/Trp ratios (43.2 vs. 36.8, *p* = 0.001, Figure 1c) and lower tryptophan concentrations (50.4 μmol/L vs. 57.9 μmol/L, *p* = 0.001, Figure 1d) were found in anemic vs. non-anemic patients. Accordingly, hemoglobin concentrations were correlated with tryptophan concentrations (rs = 0.321, *p* < 0.001) and with the Kyn/Trp ratio (rs = −0.285, *p* < 0.001). Patients with higher UICC stages presented with lower hemoglobin concentrations (I: 130 g/L, II: 138 g/L, III: 131 g/L, IV: 116 g/L, *p* = 0.003). 

In logistic regression analysis, the inflammatory markers CRP (OR 2.961 (95%CI 1.637–5.355), *p* < 0.001) and neopterin (OR 1.174 (95%CI 1.080–1.277), *p* < 0.001), as well as a higher Kyn/Trp ratio (OR 1.053 (95%CI 1.023–1.084), *p* < 0.001), were predictive for anemia—independent of sex, age, or UICC stage. Multivariate logistic regression analysis showed that Kyn/Trp was only predictive for anemia if the inflammatory markers CRP and neopterin were not included in the analysis (Z = −2.332 + 0.023 × Kyn/Trp ratio + 0.931 × CRP + 0.083 × neopterin; *p* = 0.186 for Kyn/Trp ratio, *p* < 0.001 for CRP, *p* = 0.081 for neopterin). Interestingly, in patients with elevated CRP levels, hemoglobin levels were significantly lower in those with low tryptophan concentrations (≤54.8 μmol/L) compared to those with high tryptophan concentrations (101 g/L vs. 123 g/L, *p* = 0.001).

### 3.2. Inflammation, Tryptophan, and Phenylalanine Metabolism in Patients with Solid Tumors

Neopterin serum concentrations were elevated (normal value ≤8.7 nmol/L) in 62 patients (40.8%), and 63 patients (41.4%) presented with elevated CRP concentrations (>0.5 mg/dL). Tryptophan and tyrosine concentrations were significantly lower (*p* < 0.001 and *p* = 0.021), while neopterin, kynurenine, and phenylalanine concentrations, as well as the Kyn/Trp and Phe/Tyr ratios, were significantly higher (all *p* < 0.001) in our patient cohort compared to healthy blood donors [42]. 

Women presented with higher tumor stages (*p* = 0.026); men had significantly higher hemoglobin (*p* = 0.037) and tryptophan concentrations (*p* = 0.031), and tended to have higher CRP concentrations (*p* = 0.062) and neutrophils counts (*p* = 0.064) compared to women (Table 1).

Tryptophan concentrations (I: 59.6 μmol/L, II: 58.1 μmol/L, III: 54.3 μmol/L, IV: 50.7 μmol/L, *p* = 0.090) declined with progressive UICC stages. 

### 3.3. Immune-Mediated Tryptophan Degradation and Phenylalanine Accumulation

**Figure 2:**
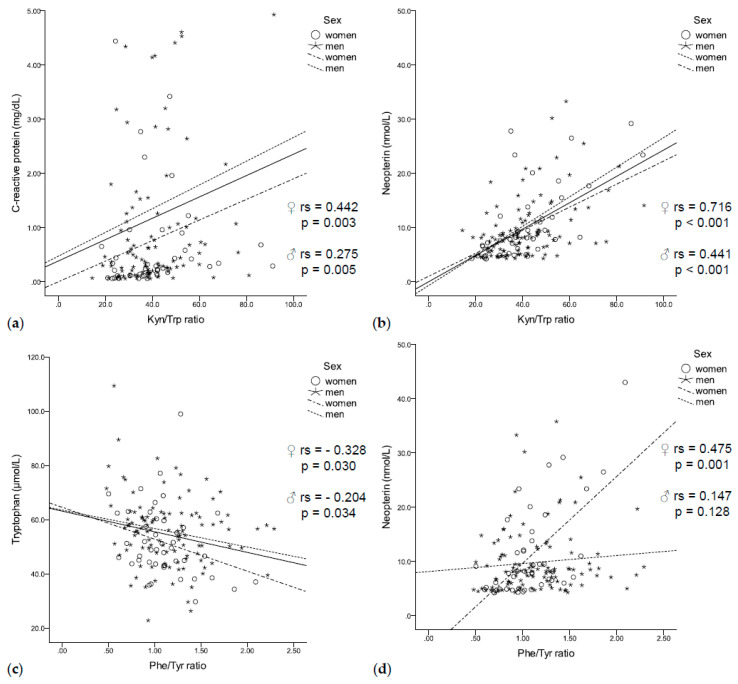
(**a**) The Kyn/Trp ratio was positively correlated with C-reactive protein (**b**) and neopterin concentrations independent of sex. (**c**) While tryptophan negatively correlated with the Phe/Tyr ratio independent of sex, (**d**) neopterin positively correlated with the Phe/Tyr ratio only in women.

In our population of patients with solid tumors, increased tryptophan degradation was associated with enhanced immune activation. High CRP concentrations coincided with increased kynurenine concentrations (rs = 0.315, *p* < 0.001) and a higher Kyn/Trp ratio (rs = 0.314, *p* < 0.001, Figure 2a), independent of sex. In addition, neopterin concentrations negatively correlated with tryptophan concentrations (rs = −0.295, *p* < 0.001) and positively correlated with kynurenine concentrations (rs = 0.413, *p* < 0.001) and a higher Kyn/Trp ratio (rs = 0.529, *p* < 0.001, Figure 2b) in men and women. 

Enhanced tryptophan degradation was further related to disturbances of phenylalanine metabolism: low tryptophan concentrations coincided with low tyrosine concentrations (rs = 0.311, *p* < 0.001) and an increased Phe/Tyr ratio (rs = –0.219, *p* = 0.007, Figure 2c). In turn, lower tyrosine concentrations (rs = –0.212, *p* = 0.009) and an increased Phe/Tyr ratio (rs = 0.232, *p* = 0.004, Figure 2d) were associated with higher neopterin concentrations. Interestingly, elevated CRP concentrations were only associated with higher phenylalanine concentrations (rs = 0.221, *p* = 0.007).

### 3.4. Quality of Life and Depression

Study participants rated their global QoL with a mean score of 61.5 ± 21.7 out of a possible maximum of 100 points. This value was lower compared to a score of 70.8 ± 22.1, which had earlier been determined as the mean score in a study of 2028 persons of the general German population [43]. Detailed results of the EORTC QLQ-C30 and the symptoms as well as functional scores are given in Table 2. 

Global QoL was related to all functioning and symptom scales except for dyspnea and financial difficulties. Interestingly, the tumor localization had no impact on the global QoL. Men and women showed no differences regarding mean QoL and symptom scales, while men had a higher physical functioning (77.4 vs. 65.9, *p* = 0.009), role functioning (65.4 vs. 51.9, *p* = 0.034), and emotional functioning (80.0 vs. 70.8, *p* = 0.041). Younger patients (age < 65 years) reported to have more pain (rs = −0.160, *p* = 0.049) and financial difficulties (rs = −0.344, *p* < 0.001), but fewer sleep disturbances (rs = 0.176, *p* = 0.031) and obstipation (rs = 0.190, *p* = 0.019) than patients of higher age. Global QoL did not significantly differ between patients with different UICC stages.

A lower global QoL was significantly related to higher BDI scores (rs = −0.545, *p* < 0.001). Similarly, all functional and symptom scales were related to BDI scores, i.e., patients with depression had more pain, complained about more fatigue, and had worse scores for emotional, cognitive, social, and physical functionality. The mean BDI score of our population was 7.5 ± 5.5 points and 61 patients (40.1%) were suffering from depression. According to the BDI-II questionnaire, 6 patients had severe depression, 15 patients moderate depression, and 40 patients minimal to mild depression. Of interest, age, tumor localization, and UICC stage had no impact on the BDI score. Interestingly, women tended to have a higher BDI score compared to men (9.1 vs. 6.9, *p* = 0.055). 

### 3.5. Relationship between Anemia, Impaired Quality of Life, Fatigue, and Depression

**Figure 3:**
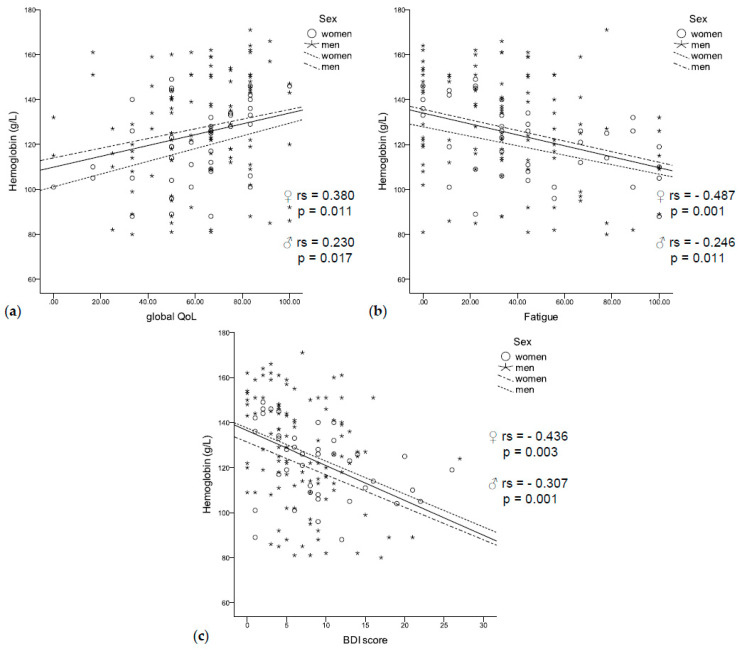
(**a**) Low hemoglobin concentrations were related to a worse quality of life, (**b**) fatigue, (**c**) and a higher BDI score independent of sex.

Anemic patients had a significantly higher BDI score (9.1 vs. 5.9, *p* < 0.001) and a significantly lower global QoL (56.5 vs. 66.2, *p* = 0.003). They reported worse physical (67.3 vs. 81.1, *p* < 0.001), social (59.4 vs. 79.2, *p* < 0.001), and role functioning (52.4 vs. 70.8, *p* = 0.001), as well as more fatigue (47.2 vs. 30.6, *p* = 0.001), sickness and vomiting (15.0 vs. 10.7, *p* = 0.042), and loss of appetite (28.6 vs. 15.1, *p* = 0.009). In accordance, low hemoglobin concentrations were correlated with a lower global QoL (rs = 0.230, *p* = 0.017, Figure 3a) and fatigue (rs = −0.321, *p* < 0.001, Figure 3b), as well as with a higher BDI score (rs = −0.307, *p* = 0.001, Figure 3c). In logistic regression analysis, higher BDI scores were related to anemia independent of sex, age, inflammation (CRP and neopterin), and UICC stage (HR 1.162 (95% CI 1.059–1.275), *p* = 0.002).

Anemic patients with low tryptophan concentrations (≤54.8 μmol/L—median) had a significantly lower global QoL (58.3 vs. 66.7, *p* = 0.014) and a higher BDI score (8.0 vs. 4.0, *p* = 0.003) when compared to non-anemic patients with low tryptophan concentrations (58.3 vs. 66.7, *p* = 0.015), while no differences were found when compared to anemic patients with high tryptophan concentrations. Anemic patients also had a significantly lower global QoL and a higher BDI score independent of a low or high Kyn/Trp ratio. 

### 3.6. Immune-Mediated Changes in Tryptophan and Phenylalanine Metabolism Are Associated with an Impaired Quality of Life, Decreased Physical Functioning, Fatigue, and Depression

**Figure 4:**
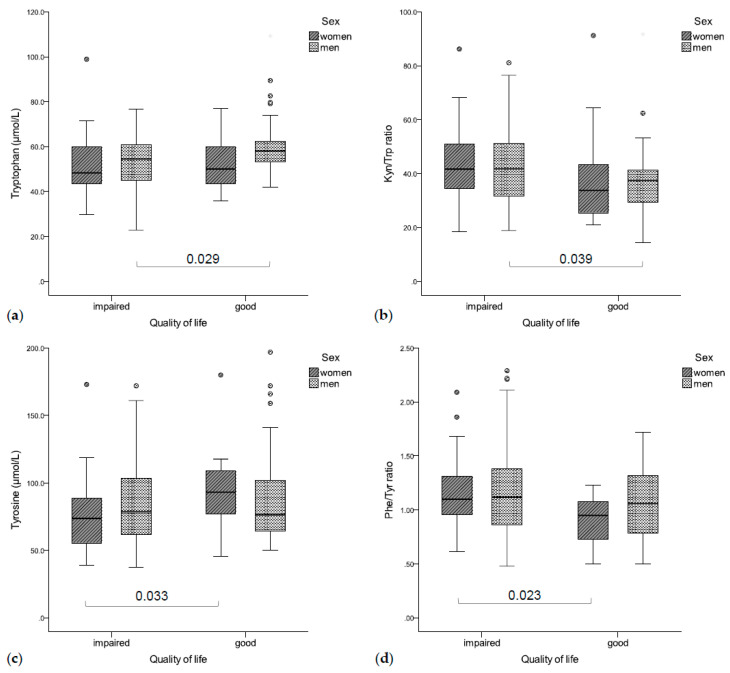
(**a**) Impaired QoL was associated with lower tryptophan concentrations (**b**) and a higher Kyn/Trp ratio (significant in men), (**c**) while women with an impaired QoL had significantly higher tyrosine concentrations (**d**) and a lower Phe/Tyr ratio than patients with good QoL.

Significant associations existed between enhanced tryptophan catabolism, inflammation, and impaired QoL, as well as increased depressive symptoms as shown in Figure 4.

Patients with impaired global QoL had significantly lower tryptophan concentrations (rs = 0.235, *p* = 0.004, Figure 4a) and an increased Kyn/Trp ratio (rs = −0.164, *p* = 0.044, Figure 4b), as well as higher CRP (rs = −0.267, *p* = 0.001) and neopterin concentrations (rs = −0.170, *p* = 0.036). In a gender-separated analysis, tryptophan concentrations were correlated with global QoL in men (rs = 0.266, *p* = 0.005), while women with a deteriorated global QoL had lower tyrosine concentrations (rs = 0.317, *p* = 0.036, Figure 4c) and a higher Phe/Tyr ratio (rs = −0.522, *p* < 0.001, Figure 4d).

Patients with a decreased physical functioning had higher Kyn/Trp (rs = −0.234, *p* = 0.004), neopterin (rs = −0.240, *p* = 0.003), and CRP concentrations (rs = −0.321, *p* < 0.001) and lower tryptophan concentrations (rs = 0.255, *p* = 0.002). In addition, fatigue was related to significantly higher CRP concentrations (rs = 0.325, *p* < 0.001) and lower tryptophan concentrations (rs = −0.189, *p* = 0.020). While the correlation with CRP was independent of sex, fatigue and tryptophan concentrations were correlated only in men (rs = −0.243, *p* = 0.012).

Depressive patients presented with higher CRP (rs = 0.169, *p* = 0.042) and neopterin concentrations (rs = 0.161, *p* = 0.048), men with more depressive symptoms presented with significantly lower tryptophan (rs = −0.230, *p* = 0.016) and higher neopterin concentrations (rs = 0.247, *p* = 0.010).

### 3.7. Therapy, Quality of Life and Depression

Patients in our study had on average 1.13 ± 0.81 previous treatments (chemotherapy, radiotherapy, and/or operation) and 82.2% had one or more pre-existing comorbidities. 

Interestingly, patients with more than one previous treatment had significantly higher hemoglobin concentrations compared to patients without pre-treatments (141 g/L vs. 121 g/L, *p* = 0.024) or only one pre-treatment (141 g/L vs. 122 g/L, *p* = 0.001). Pre-treated patients had significantly lower CRP concentrations compared to patients without previous treatments (0.33 mg/dL vs. 1.09 mg/dL, *p* = 0.008). Interestingly, those with more than one pre-treatment (*n* = 34) had significantly lower CRP (0.18 mg/dL vs. 0.44 mg/dL, *p* = 0.001), kynurenine (1.77 μmol/L vs. 2.20 μmol/L, *p* = 0.012), and tyrosine concentrations (66.5 μmol/L vs. 85.3 μmol/L, *p* < 0.001) and a lower Kyn/Trp ratio (35.8 vs. 41.1, *p* = 0.046), as well as a higher Phe/Tyr ratio (1.23 vs. 0.98, *p* < 0.001) compared to patients with one pre-treatment.

Pre-treated patients (*n* = 123, 81.5%), furthermore, had a lower global QoL (59.8 vs. 69.4, *p* = 0.034) and a higher BDI score (8.0 vs. 5.7, *p* = 0.041) compared to patients without previous treatments. They complained about having an impaired cognitive functioning (83.2 vs. 91.7, *p* = 0.048), as well as more fatigue (41.3 vs. 29.4, *p* = 0.039), pain (29.3 vs. 12.5, *p* = 0.006), and financial difficulties (22.1 vs. 6.2, *p* = 0.028). Patients with prior chemotherapy (*n* = 78, 51.7%) tended to have a higher BDI score (8.5 vs. 6.5, *p* = 0.055) compared to patients without chemotherapy.

Patients with current adjuvant or palliative anti-cancer treatment (*n* = 110, 72.4%) also had significantly lower hemoglobin concentrations compared to those without anti-cancer treatment (120 g/L vs. 144 g/L, *p* < 0.001). They reported an impaired QoL (56.9 vs. 73.4, *p* < 0.001) and had a higher BDI score (8.3 vs. 5.4, *p* = 0.001) compared to patients without current anti-cancer treatment. No significant differences were found when differentiating adjuvant (*n* = 61, 40.1%) or palliative (*n* = 49, 32.2%) treatment. Patients who were currently treated with adjuvant or palliative anti-cancer treatment had lower tryptophan concentrations (51.9 μmol/L vs. 57.8 μmol/L, *p* = 0.020) and higher CRP concentrations (0.46 mg/dL vs. 0.24 mg/dL, *p* = 0.004) as well as higher Kyn/Trp ratios (41.2 vs. 36.4, *p* = 0.018) compared to patients without current anti-cancer treatment. There was no difference regarding laboratory measurements when comparing adjuvant and palliative treatment. 

## 4. Discussion

In our study, we could show that cancer-related anemia goes along with enhanced immune-mediated tryptophan catabolism and is related to an impaired QoL and higher depression scores. Anemic patients presented with higher inflammatory markers and lower tryptophan concentrations (as well as enhanced tryptophan breakdown) compared to non-anemic patients. These data indicate that anemia might develop as a consequence of chronic inflammation, which is well-known to contribute to the development of anemia of chronic disease (ACD) [12].

Anemia was associated with reduced QoL and a tendency to depression, independent of sex, age, inflammation, and tumor stage. However, the tumor stage and anti-tumor therapy appeared to influence hemoglobin concentrations significantly: patients with higher UICC stage presented with lower hemoglobin concentrations, implying that more progressed tumor disease also impairs hematopoiesis. On the other hand, earlier treatment with chemotherapy, operations, or radiotherapy resulted in higher hemoglobin concentrations, lower inflammatory markers, and lower tryptophan breakdown compared to patients with previous anti-tumor therapy. These data indicate that anti-tumor treatment might be effective to slow down immune-mediated “complications” like anemia or disturbances of amino acid metabolism. 

However, despite these “improvements” of laboratory markers under therapy, possible side effects of anti-tumor therapy should also be taken into consideration. Patients with anti-tumor treatment often suffer from neutropenia and immunodeficiency as a consequence of chemotherapy, which strongly suppresses the bone marrow and leads to decreased myelopoiesis and hematopoiesis. Overwhelming but ineffective responses of the immune system are, thus, disrupted by chemotherapy, but whether chemotherapy is really effective to restore patients’ immune response is unclear. Additionally, patients treated with chemotherapy or radiotherapy often experience various side effects like diarrhea, pain, or dyspnea, which impair their QoL significantly. Depression and fatigue are also well-established complications of malignant disease and anti-tumor therapy; in our pilot study, 40.1% of patients presented with (mostly mild) depressive symptoms. Patients with depression had more pain, complained about more fatigue, and had worse scores for emotional, cognitive, social, and physical functionality. As all these factors influence the patients’ ability to handle everyday life and also cope with the disease, it is not astonishing that a higher load of symptoms like diarrhea or reduced appetite, fatigue, or pain also reduces patients’ QoL. Accordingly, patients with solid tumors presented with lower global QoL scores and functional scale scores as well as higher symptom scale scores compared to reference data of a German population [43].

We also found a relation between patients’ QoL, their depression tendency, and immune-mediated changes in tryptophan metabolism. Patients with lower tryptophan concentrations and a higher Kyn/Trp ratio, as well as higher neopterin and CRP concentrations, were more likely to present with a decreased physical functioning and/or fatigue. These data are in line with an earlier study in lung cancer patients, where fatigue and reduced physical functioning were seen mainly in patients with low tryptophan and higher CRP concentrations [13]. In a similar way, in patients with colorectal cancer, lower tryptophan concentrations correlated with several QoL scores [26].

Interestingly, low tryptophan concentrations and an increased Kyn/Trp ratio were related to a reduced global QoL and a higher BDI score only in men, confirming that there are gender differences. Actually, hormonal interactions with the tryptophan metabolism have been described in the literature [44]. Estrogen and progesterone have been shown to induce tryptophan breakdown via the Kyn pathway [45,46,47], which is why additional immune-mediated tryptophan degradation might not be as distinctive in women as in men. Women, furthermore, had different tumor localizations compared to men and comprised less than a third of our study population, which might also explain this finding. 

Low tyrosine concentrations and a higher Phe/Tyr ratio were related to a worse QoL only in women. Interestingly, we did not find associations between increased phenylalanine accumulation and depression in our population (neither men nor women), which is in contrast with earlier data in patients with breast cancer [48]. 

Concentrations of markers of inflammation were elevated in a high percentage of our patients (>60%) indicating that immune response against the tumor is active, but probably not effective enough to slow down tumor progression. In fact, elevated neopterin concentrations have been associated with a worse outcome in different types of cancer [49,50,51]. In addition, an increased Kyn/Trp ratio was shown to predict a worse outcome in cancer patients recently [52]. The strong correlation between elevated inflammatory markers and Kyn/Trp in our study indicates that enhanced tryptophan degradation might be primarily immune-mediated, although tumor cells are capable of inducing the enzyme IDO directly. 

The finding of a lower immune activation accompanied by a lower Kyn/Trp ratio in pre-treated patients supports this hypothesis since anti-tumor therapy was shown to affect immune activation [53]. As we, unfortunately, do not have longitudinal data of patients, we cannot infer that anti-tumor treatment inhibited overwhelming immune response and tryptophan catabolism. Further longitudinal studies in cancer patients should be conducted to address this question and to better monitor the effects of different therapies (chemotherapy, radiotherapy, surgery) on the mood, QoL, and laboratory parameters of patients. 

To conclude, our results show that anemia coincides with immune-mediated changes of amino acid metabolism in patients with solid tumors and that mood and QoL are also related to anemia. However, also other factors like anti-tumor therapy appear to affect the QoL and also the mood of patients.

### Strengths and Limitations

Results of our pilot study show for the first time that cancer-related anemia is associated with immune mediated alterations of tryptophan and phenylalanine metabolism and that anemia coincides with changes of mood and QoL in patients with different kinds of solid tumors. However, there are some major limitations of the study design, on the basis of which our results must be interpreted critically. Our study is a cross-sectional study and the population was very heterogenic. Study participants suffered from different types of cancer in different stages with different therapy strategies. Data and samples of patients who have had no earlier therapy would of course allow a better interpretation of the data, unfortunately we could not provide such data as many oncologic patients, especially those with few co-morbidities, are treated in defined clinical trials. At the time of study inclusion about 40% of patients received adjuvant therapy, 32% were under palliative therapy, only 28% did not get any therapy at the moment of study participation. Thus, we cannot rule out, that our conclusions regarding the effects of therapeutic interventions on immune activation markers and also on the mood and quality of life of patients are biased. However, our data indicate, that treatment may influence the investigated parameters, and therefore we think that further longitudinal studies with larger and more homogenous patient cohorts might provide interesting new data. It might be promising to monitor effects of different treatment regimens on immune-mediated alterations of amino acid metabolism and also focus on gender-differences of immune-mediated changes of tryptophan and phenylalanine metabolism with QoL and depression to precisely define interactions between different variables that affect the QoL of patients.

## 5. Conclusions

We could show that anemia goes along with immune-mediated changes in the metabolism of tryptophan in patients with solid tumors and is associated with depression and a deteriorated quality of life. In addition, we identified gender differences in tryptophan and phenylalanine metabolism and could confirm an association between anemia and enhanced tryptophan metabolism.

## Figures and Tables

**Table 1:** Patient characteristics and median concentrations of the investigated lab parameters *.

	Total	Women	Men	Reference
	*n* = 152	*n* = 44	*n* = 108	[42]
	Median or *n* (%)	Median or *n* (%)	Median or *n* (%)	Median
Age				
<65 years	89 (58.6)	28 (63.6)	61 (56.5)	
≥65 years	63 (41.4)	16 (36.4)	47 (43.5)	
UICC stage				
I	16 (10.8)	1 (2.3)	15 (14.3)	
II	23 (15.5)	5 (11.6)	18 (17.1)	
III	48 (32.4)	12 (27.9)	36 (34.3)	
IV	61 (41.2)	25 (58.1)	36 (34.3)	
Current treatment strategy				
Adjuvant	61 (40.1)	19 (43.2)	42 (38.9)	
Palliative	49 (32.2)	15 (34.1)	34 (31.5)	
No treatment	42 (27.6)	10 (22.7)	32 (29.6)	
Previous treatment	126 (82.9)	38 (86.4)	88 (81.5)	
Prior surgery	92 (60.5)	24 (54.5)	68 (63.0)	
Prior chemotherapy	94 (61.8)	30 (68.2)	64 (59.3)	
Prior radiotherapy	46 (30.3)	16 (36.4)	30 (27.8)	
Tumor localization				
Gastrointestinal cancer ^1^	65 (42.8)	16 (36.4)	49 (45.4)	
Lung cancer ^2^	49 (32.2)	18 (40.9)	31 (28.7)	
ENT cancer ^3^	8 (5.3)	0 (0.0)	8 (7.4)	
Sarcoma ^4^	13 (8.6)	1 (2.3)	12 (11.1)	
Tumor of other primary ^5^	17 (11.2)	9 (20.4)	8 (7.4)	
Laboratory measurements				
CRP (mg/dL)	0.37	0.29	0.46	≤0.50
WBC (g/L)	6.5	6.3	6.7	4.0–10.0
Hemoglobin (g/L)	125	122	127	≥120 (m)
				≥130 (w)
Neopterin (nmol/L)	8.2	8.0	8.2	4.96
Tryptophan (μmol/L)	54.8	48.9	56.2	66.8
Kynurenine (μmol/L)	2.07	1.96	2.10	1.72
Kyn/Trp (μmol/mmol)	38.4	38.0	38.6	26.0
Phenylalanine (μmol/L)	83.3	79.8	87.2	64.1
Tyrosine (μmol/L)	76.8	76.7	76.9	87.8
Phe/Tyr (μmol/μmol)	1.09	1.07	1.10	0.74

* Data from 152 patients are presented as median (IQR) or *n* (%) as appropriate. IQR = interquartile range; UICC = Union for International Cancer Control; ENT = ear, nose and throat; SCLC = small cell lung carcinoma; NSCLC = non-small cell lung carcinoma; CUP = cancer of unknown primary; CRP = C-reactive protein; WBC = leukocytes; Kyn = kynurenine; Trp = tryptophan; Phe = phenylalanine; Tyr = tyrosine. ^1^ Gastrointestinal (GI) cancer: esophageal cancer (*n* = 4), gastric cancer (*n* = 6), small intestine cancer (*n* = 1), colorectal carcinoma (*n* = 40), anal carcinoma (*n* = 2), hepatocellular carcinoma (*n* = 1), cholangiocellular carcinoma (*n* = 1), pancreatic carcinoma (*n* = 7), neuroendocrine carcinoma of the GI tract (*n* = 2), other gastrointestinal tumor (*n* = 1). ^2^ Lung cancer: SCLC (*n* = 9), NSCLC (*n* = 40). ^3^ ENT cancer: carcinoma of nose/paranasal sinuses (*n* = 2), oropharyngeal cancer (*n* = 4), thyroid cancer (*n* = 1), laryngeal carcinoma (*n* = 1). ^4^ Sarcoma: bone sarcoma (*n* = 3), soft tissue sarcoma (*n* = 10). ^5^ Tumor of other primary: thymic cancer (*n* = 1), pleural mesothelioma (*n* = 1), carcinoma of the adrenal cortex (*n* = 1), mamma carcinoma (*n* = 4), testicular cancer (*n* = 2), sarcomatoid carcinoma (*n* = 1), CUP syndrome (*n* = 7).

**Table 2:** Mean scores of the European Organization for Research and Treatment of Cancer Quality of Life Questionnaire Core 30 (EORTC QLQ-C30) and comparison to reference values from the general German population [43] *.

	Mean ± SD	Reference ± SD	above/below Reference Value [%]
Global health status/Quality of life	61.5 ± 22.0	70.8 ± 22.1	
Functional scales			
Physical functioning	74.1 ± 23.2	90.1 ± 16.7	63.2
Role functioning	61.5 ± 34.8	88.0 ± 22.9	68.4
Emotional functioning	77.3 ± 22.9	78.7 ± 21.0	42.1
Cognitive functioning	84.9 ± 19.9	91.2 ± 17.0	49.3
Social functioning	69.2 ± 32.3	91.0 ± 19.4	62.5
Symptom scales			
Fatigue	38.9 ± 29.9	17.1 ± 22.0	75.0
Nausea and vomiting	12.8 ± 22.1	2.8 ± 9.9	36.2
Pain	26.2 ± 30.8	15.4 ± 24.4	56.6
Dyspnea	26.9 ± 32.1	8.1 ± 20.3	50.0
Insomnia	24.3 ± 30.3	16.4 ± 27.2	46.7
Appetite loss	21.9 ± 31.9	5.4 ± 16.0	38.8
Constipation	15.9 ± 27.7	3.6 ± 13.7	30.3
Diarrhea	12.5 ± 25.4	2.8 ± 11.7	25.0
Financial difficulties	19.1 ± 30.5	6.0 ± 18.2	32.9

* Data from 152 patients are presented as means (± standard deviation) or %. High global QoL, high functional scales, and low symptom scales indicate good QoL, respectively. SD = standard deviation; QoL = Quality of life.
